# Time-of-Flight Imaging at 10 ps Resolution with an ICCD Camera

**DOI:** 10.3390/s19010180

**Published:** 2019-01-06

**Authors:** Lucrezia Cester, Ashley Lyons, Maria Chiara Braidotti, Daniele Faccio

**Affiliations:** School of Physics and Astronomy, University of Glasgow, Glasgow G12 8QQ, UK; 2400515C@student.gla.ac.uk (L.C.); Ashley.lyons@glasgow.ac.uk (A.L.); Mariachiara.braidotti@glasgow.ac.uk (M.C.B.)

**Keywords:** iCCD, LIDAR, photonics, lasers, single photon, time of flight

## Abstract

ICCD cameras can record low light events with extreme temporal resolution. Thus, they are used in a variety of bio-medical applications for single photon time of flight measurements and LIDAR measurements. In this paper, we present a method which allows improvement of the temporal resolution of ICCD cameras down to 10 ps (from the native 200 ps of our model), thus placing ICCD cameras at a better temporal resolution than SPAD cameras and in direct competition with streak cameras. The higher temporal resolution can serve for better tracking and visualization of the information carried in time-of-flight measurements.

## 1. Introduction

Time-correlated single-photon counting (TCSPC) imaging techniques are widely used for time-of-flight (TOF) imaging of ultra fast and low light events where the returning signal is correlated with the outgoing laser pulses to provide TOF information. These techniques are used in applications such as 3D ranging and underwater depth imaging [1,2,3,4,5,6] and require ultra high temporal resolution (ps resolution) and low-light capabilities (sparse photon regime) cameras e.g., single-photon-avalanche-diode (SPAD) arrays [7] or streak cameras [8] or ICCD cameras.

Streak cameras have the highest temporal resolution and can achieve temporal resolutions in the femtosecond regime [9] but in doing so are restricted to measurements in only one spatial dimension. Thus, to construct and image a 2D scene, streak cameras also require additional moving parts such as scanning elements to perform multiple measurements across the extra spatial dimension although recent implementations with additional measurements and computational processing can provide full 2D imaging capability [8].

Recently, 3D reconstruction of a target scene with millimeter scale depth resolution has also been achieved using a range-gated CMOS SPAD quanta image sensor [10].

Another widely used sub-nanosecond time-resolving camera is the ICCD. The main advantages of ICCD cameras are their wide commercial availability (compared, e.g., to SPAD cameras), low cost (compared to Streak cameras) and array format with high pixel counts (compared to both SPAD cameras and streak cameras). ICCDs are used for time-of-flight measurements, where their gating and low light detection capabilities are used for ghost imaging [11,12], underwater and 3D imaging [13,14], vehicle mounted night vision systems [15], bio-medical applications [16,17,18] and for single photon time of flight measurements [19,20] and generic LIDAR measurements [21].

These applications and others such as non-line-of-sight LIDAR for imaging behind corners rely [7,22] on the unique temporal resolution and low light detection offered by modern camera technologies such as ICCD, streak and SPAD cameras. When looking around corners, picosecond resolution is needed to be able not only to image an object’s features but also to track the object as it moves. Thus, using temporal resolution of the order of tens of picoseconds is crucial to locate hidden objects on a time scale that allows to track their trajectory [23]. Moreover, the precision of the measurements, for example, of a 3D scene, either within or outside the direct line of sight, depends directly on the temporal resolution. Furthermore, the study of some protein–protein interactions by lifetime imaging is limited by the temporal resolution of the available cameras. Currently, ICCDs with their temporal resolution of 200 ps cannot separate the components of biexponential decays [24]. Therefore, there is a constant drive towards the improvement of the temporal capabilities of these cameras [25,26,27,28,29].

We present a method that uses the time-dependent gain profile of the ICCD camera intensifier to increase the camera temporal resolution by more than a factor 20×, with a demonstrated resolution in this work of 10 ps. Compared to previous attempts to increase the temporal resolution of ICCD cameras that relied on computational processing methods that in turn required that the images and arrival times on the ICCD were sparse [30], the improvement proposed here does not require computational post-processing or any sparsity constraints and thus makes ICCD cameras a viable alternative to, for example, streak cameras. With this work, ICCD cameras become an interesting alternative for high temporal resolution (10 ps or less) imaging applications.

## 2. Method

**Figure 1:**
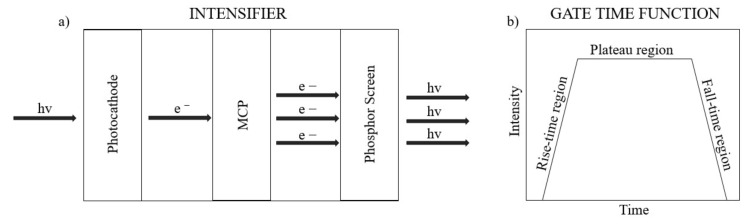
Sketch of an ICCD intensifier (**a**) and of an ICCD gate on mode function showing how voltage (directly proportional to intensity) changes with time (**b**).

The intensifier is what allows the ICCD to image low light events. It works as follows: when a photon hits the photocathode, a photoelectron is emitted. The photocathode has a certain quantum efficiency. The electron is then drawn towards a Multi Channel Plate (MCP) by an electric field (See Figure 1a).

**Figure 2:**
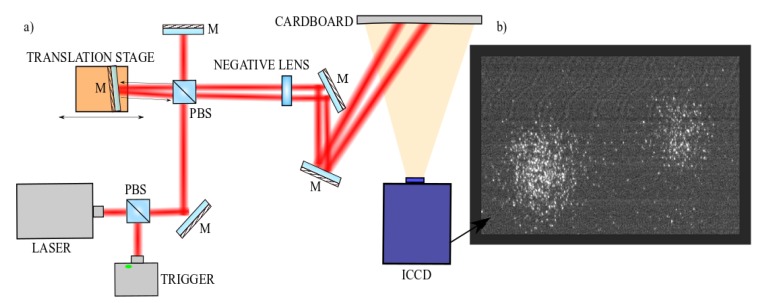
(**a**) A pulse is split with a beam splitter to hit two different mirrors, one of which is kept stationary while the other is moved by a translation stage, 3 mm at a time with respect to the first mirror. The two pulses are then redirected to pass through a diffuser and a negative lens to hit a cardboard in front of the field of view of the camera. One pulse thus hits the camera’s gate response at the same voltage value each time, while the second pulse, moved with the translation stage, hits a different voltage value each time the translation stage is moved. (**b**) The resulting image from the ICCD is displayed. One beam appears brighter/dimmer as it hits a higher/lower voltage of the camera’s gate response.

While passing through one of the channels of the MCP, the electron will undergo an avalanche process that knocks secondary electrons from the MCP with a certain gain factor. This gain factor is determined by the voltage applied to the MCP which can be selected by the user. The electrons then hit a phosphor screen that is imaged onto a CCD. The intensifier is also what allows ICCD cameras to achieve high temporal resolution. The intensifier operates on a gated ‘on’ and ‘off’ mode. During the gated ‘on’ mode, the voltage applied amplifies photoelectrons through the multi-channel plate. The shortest achievable temporal resolution of ICCD cameras is determined by the time it takes the intensifier to go from the “on” mode, in which it collects photons and amplifies the signal, to the “off” mode, in which it closes the shutter and does not allow further photons to be detected. The shortest available minimum gate time of the LaVision Picostar ICCD camera used is of 200 ps. The user can choose the voltage value of the 200 ps gate, which in turn determines the rate of multiplication of the photoelectrons. However, the voltage does not reach the desired value instantaneously, thus the minimum gate time function can be visualized as a trapezoidal function (See Figure 2b), where the flat top represents the window of time during which the voltage stays constant, while the sides comprise the time in which the voltage is changing with time. These regions where the applied voltage is changing with time will from now on be referred to as the rise-time and fall-time. By working in the rise-time or fall-time region, by imaging in the single photon regime, it is possible to improve the temporal resolution of the time of arrival of light events. The voltage determines the rate of multiplication of the photons that hit the intensifier, thus for a fixed incoming photon number when the voltage is low/high, a lower/higher intensity will be measured on the camera with the important observation that the intensity is directly correlated to the arrival time on the intensifier (and not to the incident photon number). In other words, since the voltage, during the rise and fall time of the camera’s opening gate, is changing with time, the measured intensity values change depending on at which point of the rise/fall-time the light event hits the camera gate. We exploit the change of voltage with time to assign a time of arrival to photons with a resolution of 10 ps, restricted to light events that arrive during the rise or fall-time of the camera gate “on” mode. This method therefore allows a temporal resolution to be achieved that is significantly improved with respect to the native gating time of the camera when working in the single photon regime. A filter can be applied to make sure to attenuate the incoming light as required. By changing the delay time at which the ICCD starts its acquisition, it is possible to image scenes with depth greater than the distance light travels during the rising time of the camera.

## 3. Experimental Set-Up

The laser source used to illuminate a scene and measure the camera temporal resolution is a Ti:Sapphire oscillator with a pulse duration of 140 fs with a repetition rate of 80 MHz and a center wavelength of 810 nm. The ICCD camera used is a LaVision Picostar, with a minimum temporal gate (“on” mode) of 200 ps for. The quantum efficiency is 8%. The maximum repetition rate of the intensifier gate voltage is 1 MHz whereas the actual CCD has a minimum acquisition time of 20 ms.

For our measurements, we acquired an image for 110 different delays (in 10 ps steps) between the femtosecond pulse and the intensifier. A trigger from the laser is used to synchronize the camera to the laser pulses.

We imaged two attenuated pulses hitting a cardboard in front of the field of view of the camera, with a controllable delay that was changed in steps of 10 ps (See Figure 2). We attenuated the laser pulse with a neutral density filter down to the single photon level. Indeed, the goal is to use the rising edge time-varying gain to associate an intensity on the CCD to an arrival time of the laser pulse. This requires the use of a fixed number of photons so as to avoid any CCD intensity variations due to input light intensity variations. The simplest route to this is to therefore work in the photon-starved regime where much less than 1 incident photon per pixel is recorded on the camera (e.g., less than 0.01 photons per laser pulse, per pixel). In this regime, we have close to 100% certainty that each intensity recording on the CCD plane is due to a single incident photon and therefore, any variations in the measured intensity are due to the photon arriving at a different time and thus experiencing a different gain.

## 4. Temporal Response of the ICCD Camera

**Figure 3:**
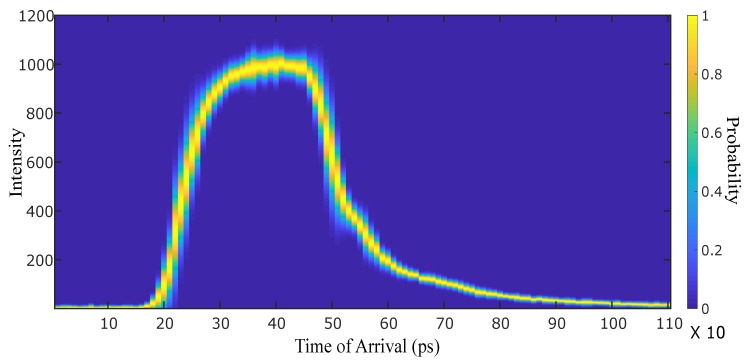
Estimation of the camera temporal response and jitter. Given a specific intensity value on the rising or falling edge, the most likely time of arrival can be obtained to within the standard deviation of the curve (i.e., the full width of the curve thickness) that is 10 ps for our ICCD camera.

Our first objective was to calibrate the camera to obtain its temporal response. The ICCD objective was illuminated uniformly using 110 time delays separated by 10 ps steps and this process was repeated 65 times so as to retrieve statistics on the temporal jitter and precision with which the time gate delay can be fixed. Indeed, it it is necessary for the proposed technique that the temporal instant at which the intensifier’s shutter opens is at a fixed time from one acquisition to the next. However, some temporal jitter is to be expected, due to the camera’s electronics, and this will ultimately limit the maximum precision achievable. Indeed, for each time delay we constructed the intensity distribution in time with a set of 65 measurements finding a Gaussian function with a FWHM of about 10 ps in concordance to the value of the electronic jitter provided by LaVision. This is therefore the main limiting effect in our measurements, i.e., from only the measured intensity for a fixed input photon number, it is possible to infer the time-of-arrival to within 10 ps certainty, which sets our temporal delay. At each temporal delay, for each pixel of the ICCD, we calculated the intensity mean and standard deviation from the intensities recorded during the 65 repetitions. This allows to construct for each pixel a probability density function (PDF) of the measured intensity as a function of the gate delay. Figure 3 shows the PDF obtained for a single pixel (all other pixels exhibited essentially identical behaviour, small differences are attributed to time delays between pixels due to the MCP response, however these time delays are within the order of magnitude of the jitter). The color map represents the probability of the photons’ arrival time for a given intensity value.

Finally, it should be noted that this can only be done under the assumption that the duration of the measured event (here our pulses had a duration of 140 fs) is much shorter than the temporal rise time of the detector (80 ps).

## 5. Single Photon Arrival Time Measurements

In order to map CCD intensities to arrival times, we need to fix the number of photons. Our choice was to work in the photon starved regime (i.e., only single photons/pixel are incident on the ICCD), which is also of interest for a number of applications, including LIDAR, non-line-of-sight imaging where the return signal can be extremely weak.

Our set-up for this experiment is shown in Figure 2. At first, we characterized the background by acquiring data while the beam is off. A histogram was thus generated of the CCD pixel intensities for the background signal and a Gaussian function was found to be the best fit (with a peak centred at 4 counts and FWHM also of 4 counts).

**Figure 4:**
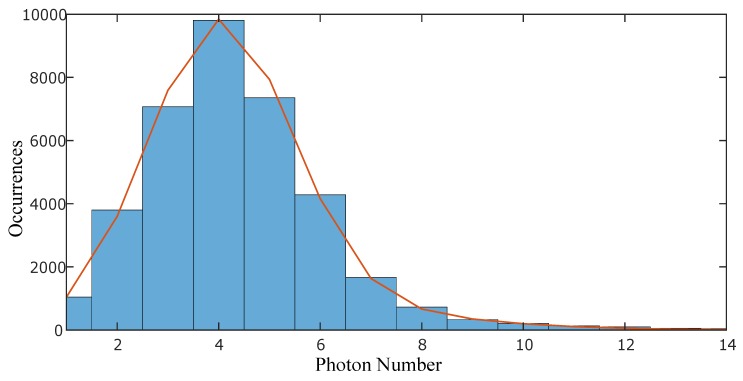
Histogram of intensity counts for a pulse arriving at a fixed value of the camera’s rise time. The histogram is fitted with a function which is a summation of a Gaussian and a Poisson function. The Gaussian function is due to the background while the Poisson distribution is due to the sparse photon counts of the signal.

Then, we acquired data with the laser. The translation stage was first positioned so that the two pulses hit the rising gradient at the same time, enabling the pulses to be amplified by the ICCD hitting the gate at the midpoint of the rising gradient. Then, one pulse was kept stationary while the other pulse delay was varied in steps of 10 ps (from −20 ps to +20 ps) by moving the translation stage by 3 mm each time. We then created separate histograms for the two pulses at each time frame. These histograms were found to be best fitted with a function that was the sum of a Gaussian (representing the background noise, characterized in the first step) and a Poisson distribution (See Figure 4).

**Figure 5:**
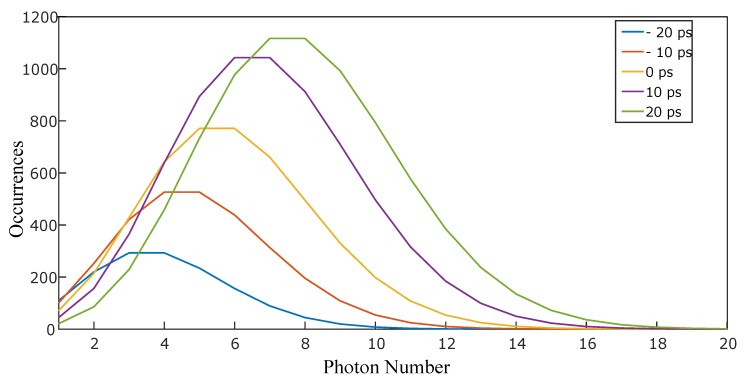
The Poisson distributions have been extrapolated from the Gaussian plus Poisson function fitted to the histograms of photon counts of the laser beam.

**Figure 6:**
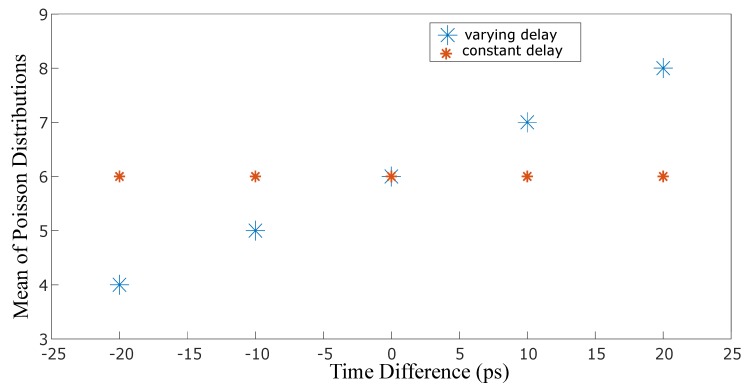
A graph of intensity vs time difference between the two pulses. One pulse hits the rising gradient at the same voltage value, thus having the same intensity each time (small fluctuations due to the quantum efficiency of the intensifier). The second pulse is moved by the translation stage, by 3 mm at a time, thereby arriving at different regions of the rising gradient.

In Figure 5, we show the values of the single free parameter of the Poisson fit 〈N〉 (the value of the photo-electron counts on the CCD) for each delay. As can be seen from Figure 6, where the mean values of the Poisson distributions have been extrapolated, 〈N〉 follows a clear linear relation across the delay range tested in our measurements, thus indicating that by simply characterizing the Poisson distribution of photon detection events in each region of the camera, it is possible to precisely determine, to within 10 ps, the arrival time of the photons in that region.

## 6. Conclusions

We have shown that by using the gain-dependent amplification in the rising edge of the ICCD intensified gate, it is possible to map the average photo-electron counts on the ICCD, 〈N〉, to an arrival time on the camera that is limited only by the 10 ps electronic jitter of the camera. This is in contrast with standard sliding-gate techniques that rely on the overall ICCD gate time to achieve temporal resolution and that are thus limited to 200 ps or more.

This improvement of ICCD temporal resolution down to 10 ps makes these cameras an effective alternative to streak cameras, albeit without the requirement for further scanning or computational approaches to achieve full 2D imaging.
